# Overexpression and purification of U24 from human herpesvirus type-6 in *E. coli*: unconventional use of oxidizing environments with a maltose binding protein-hexahistine dual tag to enhance membrane protein yield

**DOI:** 10.1186/1475-2859-10-51

**Published:** 2011-06-29

**Authors:** Andrew R Tait, Suzana K Straus

**Affiliations:** 1 Department of Chemistry, University of British Columbia, 2036 Main Mall, Vancouver, BC, V6T 1Z1, Canada

## Abstract

**Background:**

Obtaining membrane proteins in sufficient quantity for biophysical study and biotechnological applications has been a difficult task. Use of the maltose binding protein/hexahistidine dual tag system with *E.coli *as an expression host is emerging as a high throughput method to enhance membrane protein yield, solubility, and purity, but fails to be effective for certain proteins. Optimizing the variables in this system to fine-tune for efficiency can ultimately be a daunting task. To identify factors critical to success in this expression system, we have selected to study U24, a novel membrane protein from Human Herpesvirus type-6 with potent immunosuppressive ability and a possible role in the pathogenesis of the disease multiple sclerosis.

**Results:**

We expressed full-length U24 as a C-terminal fusion to a maltose binding protein/hexahistidine tag and examined the effects of temperature, growth medium type, cell strain type, oxidizing vs. reducing conditions and periplasmic vs. cytoplasmic expression location. Temperature appeared to have the greatest effect on yield; at 37°C full-length protein was either poorly expressed (periplasm) or degraded (cytoplasm) whereas at 18°C, expression was improved especially in the periplasm of C41(DE3) cells and in the cytoplasm of oxidizing Δtrx/Δgor mutant strains, Origami 2 and SHuffle. Expression of the fusion protein in these strains were estimated to be 3.2, 5.3 and 4.3 times greater, respectively, compared to commonly-used BL21(DE3) cells. We found that U24 is isolated with an intramolecular disulfide bond under these conditions, and we probed whether this disulfide bond was critical to high yield expression of full-length protein. Expression analysis of a C21SC37S cysteine-free mutant U24 demonstrated that this disulfide was not critical for full-length protein expression, but it is more likely that strained metabolic conditions favour factors which promote protein expression. This hypothesis is supported by the fact that use of minimal media could enhance protein production compared to nutrient-rich LB media.

**Conclusions:**

We have found optimal conditions for heterologous expression of U24 from Human Herpesvirus type-6 in *E.coli *and have demonstrated that milligram quantities of pure protein can be obtained. Strained metabolic conditions such as low temperature, minimal media and an oxidizing environment appeared essential for high-level, full-length protein production and this information may be useful for expressing other membrane proteins of interest.

## Background

U24, a membrane glycoprotein from Human Herpesvirus Type-6A (HHV-6A), has garnered recent interest because a N-terminal fragment of the protein was shown by Tejada-Simon *et al*. to activate T-cells [1], and cause them to cross-react with myelin basic protein, an autoantigen targeted in the pathogenesis of multiple sclerosis (MS). Sullivan *et al*. showed that *in vivo *expression of U24 alone could downregulate CD3 T-cell receptor and transferrin receptor cell-surface expression, and impair T-cell activation [2,3]. We have previously demonstrated U24 as an *in vitro *kinase target for ERK2 MAP kinase, further implicating a potential role for U24 in immune-modulating activity [1,4,5]. Using the TMMH Server Version 2 [6], open reading frame analysis for U24 from HHV-6A (Strain U1102) predicts expression of an 87 amino acid, 10 kDa glycoprotein, identified to have a single transmembrane pass (residues 57-79). U24 also has two cysteines (Cys21 and Cys37), whose oxidation state is unknown.

Structure/function studies of membrane proteins of biological interest such as U24 has historically been a difficult task, primarily because of the limited amount of material available [7]. Proteins extracted from natural sources can also have a backdrop of post-translational modifications such as glycosylation, phosphorylation, etc., thereby precluding any meaningful structural study of the heterogeneous pool of protein. It was demonstrated that U24 appeared to have extensive post-translational modifications if expressed in human cells, consistently giving molecular weights of 20 and 23 kDa by SDS-PAGE [3], which is more than double the mass predicted by the primary sequence. Heterologous expression of U24 in a prokaryotic system such as *E. coli *can therefore represent a cost-effective and relatively easy way to obtain large yields of homogeneous membrane proteins where post-translational modifications can be added subsequently in a controlled manner.

Protein fusion tags have a direct bearing on expression levels, solubility and/or efficiency of purification. Typical fusion tags used include glutathione-S-transferase (GST) [8], maltose binding protein (MBP) [9] and hexahistidine (6 × His) tags [10], amongst many others. Since no single bacterial strain or fusion tag has been shown to work in all cases for membrane proteins, the trial-and-error task of discovering which selection will work to produce enough pure protein for study can be quite daunting [11]. In a high-throughput screening sense, an approximate yield of 0.5 mg of purified membrane protein per L of *E.coli *culture has been considered the acceptable lower expression limit for cost-effective scale up with the intent on performing structural studies [12]. A yield of 3-5 mg of purified membrane protein per L of culture is deemed to be a high level [13].

Combining the solubility-enhancing chaperone ability of MBP [9,14] with the high affinity of a polyhistidine tag (≥ 6 consecutive histidines) for efficient protein expression and purification is not a recent development [15], but has seen a recent revival in the context of high-throughput protein production [16]. Even so, the combinatorial use of MBP and 6 × His tags is only beginning to be extensively explored with membrane proteins. For instance, Korepanova *et al*. demonstrated that this system could enable a 70% success rate in expressing 16 of 22 integral membrane proteins from *Mycobacterium tuberculosis *in the cytoplasm of *E. coli*, proteins which would otherwise be unobtainable due to poor or undetectable expression levels [17].

An added benefit of using MBP as a fusion tag is that one can generally choose the location within the *E. coli *where the expressed protein resides: either in the cytoplasm or periplasm [16]. MBP contains a signal sequence and thus naturally resides in the periplasm of *E. coli*, yet if the signal sequence is deleted, MBP remains in the cytoplasm. While the most common approach is to express fusion proteins in the cytoplasm [16], because the yield is generally higher than those expressed in the periplasm, there may still be valid reasons why the expression of a protein of interest should be directed to the periplasm. For instance, the guinea pig sigma-1 receptor, when engineered as a fusion to the MBP-6 × His dual tag, could only be expressed as a periplasmic-directed fusion protein [18]. In this case, it was hypothesized that the receptor portion becomes stably embedded in the *E. coli *membrane as a result of the periplasmic transport process. This hypothesis is consistent with what was observed for the expression of the aquaporin Z membrane protein as a periplasmic MBP fusion, where 200 mg/L of protein were obtained, but was not found to be secreted out into the periplasmic space [19].

If there are cysteines in the primary sequence of a protein and their oxidation state is unknown, as is the case with U24, it can be beneficial to express the protein in the oxidizing environment of the periplasm [20] or use an oxidizing strain of *E.coli *[21]. It is generally accepted that disulfide bonds cannot be formed in the cytoplasm of *E. coli *unless strains such as Origami (Novagen), which are defective in reductases (*(Δtrx/Δgor) *are used. More recently however, it was shown that disruption of reducing pathways in *E.coli *was not absolutely essential to obtain disulfide-bonded proteins in the cytoplasm. Introduction of the sulfhydryl oxidase Erv1p into a native reducing cytoplasm was used to achieve greater yields of a disulfide-bonded protein than with reductase-deficient strains alone [22,23].

Interestingly, MBP may help promote correct disulfide bond formation in fused proteins. Planson *et al*. [24] showed that the C-terminal fragment of *Plasmodium falciparum *merozoite surface protein 1 was misfolded and formed disulfide linked polymers when expressed untagged in the periplasm, but was a correctly folded monomer with six intramolecular disulfides when expressed fused to MBP. This result clearly demonstrates the potential that MBP has for introducing correct disulfide bonding into globular proteins, a property which would be important in membrane proteins as well.

In this study, we demonstrate how using a fusion to a maltose binding protein/hexahistidine tag can be beneficial for the expression of good quantities of pure U24. This work also examines the effects of temperature, growth medium type, cell strain type, oxidizing vs. reducing conditions and periplasmic vs. cytoplasmic expression location on expression yields.

## Results

### Cloning and Expression

**Figure 1:**
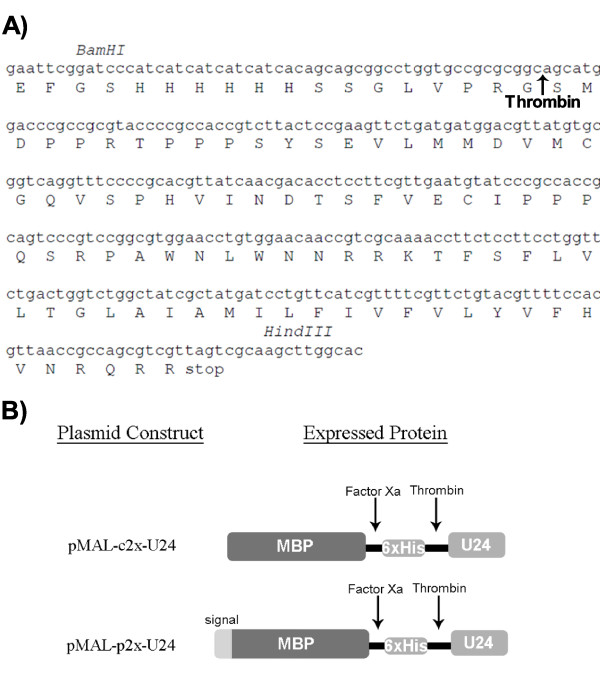
**U24 codon-optimized gene, amino acid sequence and graphical representation of expressed protein construct**. A) *BamHI/HindIII *cut sites are indicated and used to clone the PCR-amplified duplex DNA into the corresponding sites of pMAL-p2x and pMAL-c2x vectors, from which the MBP-6 × His-U24 fusion protein is expressed. The U24 gene was designed to be preceded by a hexahistidine tag (6 × His) and LVPRGS thrombin cleavage site (indicated by an arrow). Final thrombin-cleaved and purified U24 protein will include an additional two amino acids (Gly-Ser) at the N-terminus. B) Cartoon representation of expressed protein. The difference in constructs is a signal sequence at the N-terminus of the protein expressed by pMAL-p2x-U24, directing expression to the periplasm. The Factor Xa cleavage site is vector-encoded.

Oligonucleotide fragments representing the U24 gene were synthesized and the full-length gene was obtained by overlap PCR, before being cloned into two expression vectors: pMAL-c2x, pMAL-p2x (New England Biolabs) (Figure 1). Vectors pMAL-c2x and pMAL-p2x direct the MBP-6 × His-U24 fusion protein to the *E. coli *cytoplasm and periplasm, respectively. The commercially available *E. coli *cell strains that were chosen for expression were XL1-Blue (Stratagene), BL21(DE3) (Stratagene), C41(DE3) (Lucigen), Origami 2 (Novagen), and SHuffle (New England Biolabs). The C41(DE3) strain [25] is a derivative of the commonly used BL21(DE3) strain which allows high expression yields of membrane proteins by reducing their toxicity to the host [11]. Origami 2 is deficient in the glutathione and thioredoxin reductases (Δ*trx/Δgor*) and therefore provides an oxidizing environment conducive to disulfide formation. SHuffle is deficient in the same reductases, but expresses a signal-truncated disulfide bond isomerase (DsbC) in the cytoplasm. Normally found in the periplasm, DsbC rearranges incorrect disulfide bonds [26] and can also act as a chaperone to assist in the proper folding of proteins that do not require disulfide bonds [20].

**Figure 2:**
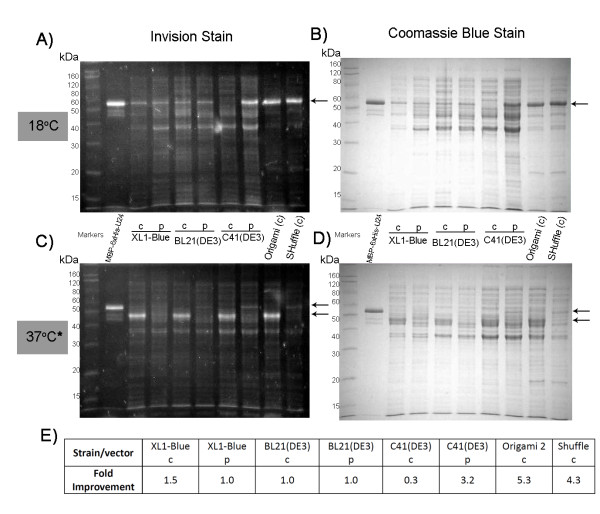
Comparison of MBP-6 × His-U24 expression in various *E. coli *strains, at low (18°C) and high temperature (37°C*). Cultures induced at 18°C (A&B) and 37°C* (C&D) and visualized with His-tag-specific Invision stain (A&C) and Coomassie Blue stain (B&D). Cytoplasmic and periplasmic expressions are denoted by c and p, respectively. While at high temperature (C&D) MBP-6 × His-U24 appears degraded (c lanes) or poorly expressed (p lanes), optimal expression conditions are observed at low temperature especially in the periplasm of C41 (DE3), and cytoplasm of Origami 2 and SHuffle. (*)SHuffle was grown at 30°C, as per vendor's recommendations, perhaps explaining why at this lower temperature some trace full length MBP-6 × His-U24 is observed. Position of full-length MBP-6 × His-U24 (right of the marker lane) is denoted by arrows in A&B, upper arrow in C&D (lower arrows point to truncated/degraded protein). Summary of fold-enhancement for full-length MBP-6 × His-U24 expression at 18°C in the various strains/vectors is listed in E), based on densitometric analysis of bands represented A&B, and normalized against corresponding BL21(DE3) cytoplasmic/periplasmic expression.

Our attempts to express MBP-6 × His-U24 in the cytoplasm of various cell lines at 37°C yielded a protein that was truncated (Figure 2C&Figure 2D, indicated by lower arrow). This truncated product could be easily purified by Ni^2+ ^affinity and gave a mass of ~45 kDa by MALDI-TOF MS (data not shown), suggesting that this isolated form is the MBP-6 × His tag with most of the U24 portion absent. Of the numerous parameters we tested, lowering the induction temperature to 18°C appeared to have the greatest effect on alleviating the problem of protein truncation, yet the expression of full-length protein was still somewhat poor in the cytoplasm of XL1-Blue, BL21(DE3) and C41(DE3) strains (Figure 2A&Figure 2B, indicated by arrow). When expression of MBP-6 × His-U24 was directed to the periplasm, low levels of full-length protein could be observed at either 18°C or 37°C, but a substantial increase in yield of the full-length protein was observed for C41(DE3), especially over either the XL1 Blue or BL21(DE3) strains. Densitometric analysis suggest that periplasmic MBP-6 × His-U24 expression in C41(DE3) at 18°C is 3.2 × greater than in BL21(DE3) when expressed under identical conditions (Figure 2E). An interesting yet unexplained phenomenon is that cytoplasmic expression of MBP-6 × His-U24 in C41(DE3) is actually 3 × less than BL21(DE3) at 18°C. It is unclear why C41(DE3) should fare any better (or worse) than its parent strain BL21(DE3), since C41(DE3) is presumed to only enhance protein production from a T7-based promoter [11]; here a P_tac _promoter was used. We were prompted to question whether it was specifically an oxidizing environment like the periplasm that had a positive effect on expression levels. When MBP-6 × His-U24 was expressed in the oxidizing cytoplasm of Origami 2 and SHuffle, higher expression was observed in these strains over the reducing cytoplasm of the other strains tested. Densitometry revealed that MBP-6 × His-U24 expression is an estimated 5.3 × higher in Origami 2 and 4.3 × higher in SHuffle compared to BL21(DE3) (Figure 2E). Low temperature was still critical for the generation of full-length protein. The presence of DsbC in the SHuffle strain did not enhance the apparent protein yield over Origami 2 cells. Since MBP has no cysteines, these findings suggested that the oxidation state of the cysteines in U24 may have an effect on expression levels. The chaperone-like qualities of the MBP moiety may help protect the U24 passenger protein against proteolysis under oxidative conditions and low temperature, enabling optimal conditions for folding and possible disulfide-formation. An alternative factor for increased yields is that degrading proteases are either inactive or absent under the oxidizing, low-temperature expression conditions that were tested.

### Purification and Isolation of U24

**Figure 3:**
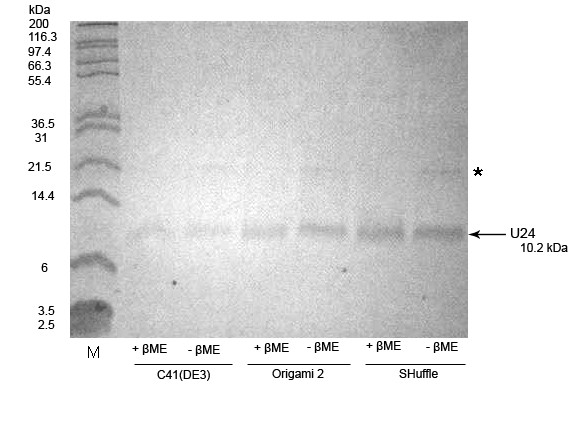
**SDS-PAGE analysis of U24 purified from C41(DE3), Origami 2 and SHuffle *E.coli*, in the presence and absence of β-mercaptoethanol reducing agent**. Isolated U24 was from indicated strains, cultured in LB media. Protein solution was mixed 1:1 (vol:vol) with 2X SDS-PAGE buffer ± 2.5% β-mercaptoethanol (βME) final concentration, and analysis carried out by Tris-Tricine SDS-PAGE. Protein amounts were approximately as follows: U24_C41(DE3) _, 0.1 μg; U24_Origami2 _and U24_SHuffle_, 0.4 μg. M: molecular markers. Arrow points to location of monomeric U24 (10.2 kDa); * indicates U24 dimer.

MBP-6 × His-U24 expressed in the C41(DE3) periplasm, Origami 2 and SHuffle cytoplasm at low temperature were extracted in soluble form and purified by Ni^2+^-affinity (Histrap) chromatography. After digestion by thrombin, U24 was liberated from MBP-6 × His and further purified to homogeneity by applying the digest mixture to tandem Histrap and Q-sepharose columns, and collecting U24 in the flowthrough. U24 protein that had been purified from the three *E. coli *strains ran as a monomer under both reducing and non-reducing conditions, indicating that U24 does not form significant amounts of disulfide-linked polymers (Figure 3), although some trace amounts of what is presumed to be a U24 dimer was observed under some conditions. The dimer disappears upon addition of reducing agent. Since our recombinant form of U24 runs at ~10 kDa by SDS-PAGE, we conclude that we have avoided the unknown post-translational modifications which cause U24 to run as a doublet of 20 and 23 kDa when expressed from human cells. Highest yields of U24 (Table 1) were from C41(DE3) grown in M9 minimal media. Pryor *et al*. [15] suggested that use of minimal media reduces the levels of endogenous *E.coli *proteases which cleave the sensitive region between the MBP-6 × His tag and protein of interest.

**Table 1:** Expression summary and purification yields for cultures grown at 18°C

Vector	Expressed protein	Cellular location	C41(DE3) *E.coli *isolated U24 (mg/L)	Origami 2 *E.coli *isolated U24 (mg/L)	Shuffle *E.coli *isolated U24 (mg/L)
pMAL-c2x	MBP-6 × His-U24	Cytoplasm	N/A	1.84 ± 0.31	1.60 ± 0.24

pMAL-p2x	MBP-6 × His-U24	Periplasm	0.46 ± 0.27 2.81 ± 0.32 (M9)	N/A	N/A

Results are based on the intensity of expected bands observed by SDS-PAGE and by the BCA assay. U24 was purified as described from the indicated strains grown in 4 × 1 L of LB (or M9), induced at 18°C. N/A: Not applicable. Concentration is the mean ± s.d. of n = 4-6 independent assay measurements.

### Secondary Structure Analysis of Purified U24 by CD Spectroscopy

**Figure 4:**
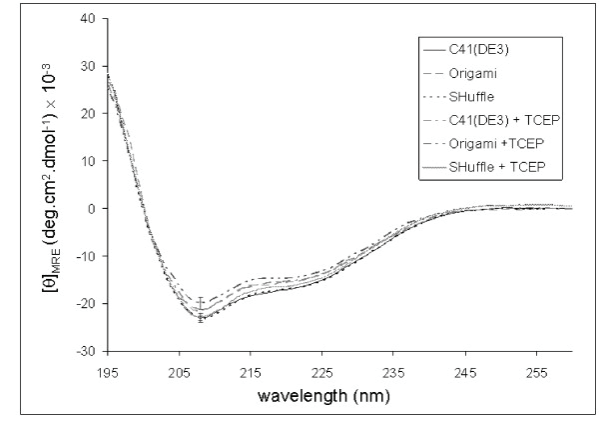
**Far-UV CD spectra of U24 obtained from the different cell strains used in this study**. U24 protein that had been isolated from various *E. coli *cell strains was reconstituted in 10 mM Tris·HCl, 10 mM NaCl, 10 mM SDS, pH 7.5 and far-UV CD was run in the presence and absence of tris(2-carboxyethyl)phosphine (TCEP) reducing agent. Concentration of protein in the samples were determined by BCA assay to be [U24]_C41(DE3) _= 0.106 ± 0.004 mg/ml, [U24]_Origami _= 0.093 ± 0.006 mg/ml, and [U24]_SHuffle _= 0.118 ± 0.004 mg/ml, where the standard deviation is based on four independent assay measurements.

**Figure 5:**
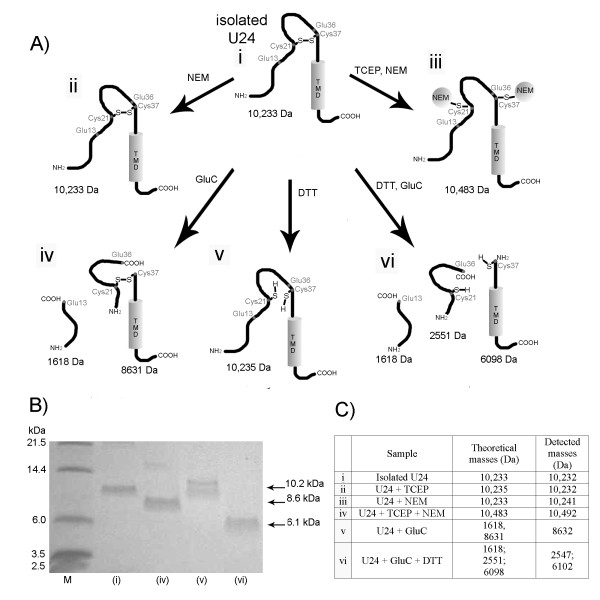
**Characterization of the disulfide in isolated U24 protein**. A) Illustration of isolated U24 protein, demonstrating the formation of a single intramolecular disulfide bond between the only two cysteines in the molecule (i). N-ethylmaleimide (NEM) does not react with the oxidized, disulfide bonded protein (ii) and NEM only modifies cysteines in the reduced form (+125 Da per cysteine) once they are reduced by tris(2-carboxyethyl)phosphine (TCEP) (iii). Use of glutamyl endoproteinase (GluC), which cleaves peptide bonds primarily after glutamic acid under these conditions, yielded two fragments if U24 contained an intramolecular disulfide (iv). Using dithiothreitol (DTT) to reduce the disulfide in U24 (v), GluC cleavage then gave three proteolytic fragments for U24 (vi). B) Tris-Tricine SDS-PAGE results of U24 (isolated from SHuffle) ± DTT and GluC-digested. M: molecular markers; oxidized U24 ((i), 10.2 kDa) shifts to a lower mass once cleaved ((iv), 8.6 kDa) and U24 reduced by DTT ((v), 10.2 kDa) shifts to even lower mass when cleaved ((vi), 6.1 kDa). C) MALDI TOF MS analysis of U24 modified with NEM ± TCEP, and cleaved by GluC ± DTT. Tabulated theoretical masses represent U24 species indicated in A) (i-vi). Experimental masses that were detected are ± 0.2% the theoretical mass.

In order to determine whether the strains used have an impact on secondary structure of the purified protein, we determined the structure of the purified U24 obtained above in the presence of membrane-mimetic SDS detergent, observed by circular dichroism (CD) (Figure 4). U24 was found to be highly α-helical under all conditions tested (Table 2). The relative proportions of secondary structure components (α-helix. β-sheet, etc.), as determined by fitting the spectra in Figure 5 using the programs CONTILL, SELCON3, and CDSSTR [27], remain highly similar in the presence and absence of TCEP reducing agent.

**Table 2:** Secondary Structure Analysis of Purified U24 by CD Spectroscopy

U24 sample^a^	Secondary Structure Fraction^b^						NRMSD^c^
	**α_R_**	**α_D_**	**β_R_**	**β_D_**	**T**	**U**	

C41(DE3)	0.412	0.208	0.006	0.032	0.113	0.234	0.150

C41(DE3) + TCEP	0.367	0.212	0.018	0.035	0.127	0.243	0.143

Origami	0.379	0.223	0.023	0.032	0.120	0.222	0.130

Origami + TCEP	0.341	0.201	0.021	0.041	0.134	0.256	0.130

SHuffle	0.411	0.204	0.003	0.03	0.117	0.231	0.156

SHuffle + TCEP	0.401	0.212	0.013	0.032	0.112	0.239	0.147

^a^U24 protein that had been isolated from various *E.coli *cell strains was reconstituted in 10 mM Tris·HCl, 10 mM NaCl, 10 mM SDS, pH 7.5 and far-UV CD was run (260-195 nm) in the presence and absence of tris(2-carboxyethyl)phosphine (TCEP) reducing agent.

^b^Fractions of secondary structure were determined by combining the software analysis results of the CONTIN/LL, SELCON3 and CDSSTR programs [27] using the SMP56 reference data set. Structural classes are given as: α_R, _regular α-helix; α_D_, distorted α-helix; β_R_, regular β-strand; β_D_, distorted β-strand; T, turns; U, unordered.

^c^Normalized Root-Mean-Square Deviation. NRMSD is defined as Σ[(θ_exp _- θ_cal_)^2^/(θ_exp_)^2^]^1/2^, summed over all wavelengths, and where θ_exp _and θ_cal _are, respectively, the experimental ellipticities and ellipticities of the back-calculated spectra for the derived structure [27,35]. It has been suggested [36] that experimental and calculated spectra are in good agreement if NRMSD < 0.1, are similar if 0.1 < NMRSD < 0.2, and are in poor agreement if NRMSD > 0.2.

### Disulfide Analysis

MALDI-TOF MS was used to analyze purified U24 obtained from the three *E.coli *strains representing the highest overexpressed proteins levels, confirming that U24 existed in a monomeric form of expected molecular mass (experimental = 10,235 ± 0.2% [4]). When the cysteine modifying reageant N-ethyl maleimide (NEM) was added alone, no increase in mass was observed. NEM cannot directly react with disulfide-bonded cysteines. If a reducing agent is added such as tris(2-carboxyethyl)phosphine (TCEP), the cysteine disulfide in U24 is broken, and then the mass shift attributed to both cysteine thiols being modified by NEM could be observed (+250 m/z) (Figure 5A&Figure 5C). These results are the same for U24 samples obtained from all strains tested. To further confirm the presence of the disulfide between Cys21-Cys37, a sample of U24 from SHuffle was subjected to GluC protease digestion in the presence and absence of reducing agents, and the resulting peptide fragments were analyzed by MALDI-TOF MS and SDS-PAGE (Figure 5B&Figure 5C).

### Analysis of Cys-free U24 to Determine the Effects of the Disulfide on Protein Expression

**Figure 6:**
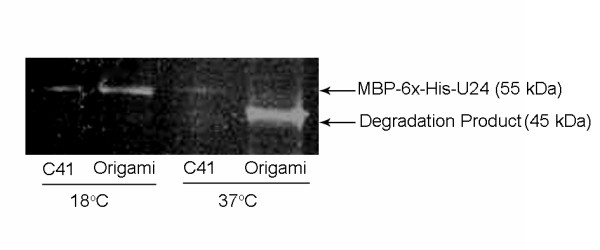
**Examination of cysteine-free mutant U24 expression**. The C21SC37S mutant U24 constructs were expressed: pMAL-p2x-U24 in C41 (DE3) and pMAL-c2x-U24 in Origami 2 cells, at 18°C and 37°C. Removal of disulfide bond potential appeared to have no effect on *in vivo *stability of expressed MBP-6 × His-U24, which exhibited the same expression characteristics as wild-type; a similar loss in mass of the degraded fusion protein is observed at the higher temperature.

After confirming that our recombinant U24 forms an intramolecular disulfide bond, another objective was to see if formation of this bond directly contributed to the stability of the expressed full-length MBP-6 × His-U24. When Cys-free C21SC37S mutant U24 plasmids were expressed under ideal periplasmic and cytoplasmic conditions (pMAL-p2x-U24 in C41(DE3) and pMAL-c2x-U24 in Origami 2) in LB media, we observed a similar trend in expression as compared to wild-type by SDS-PAGE (Figure 6 and 2A). Removal of the disulfide bond had no apparent effect on the ability to produce full-length MBP-6 × His-U24, neither promoting degradation at low-temperature nor enhancing yield of full-length at high temperature.

### Comparison of Cellular Mass Yields

**Figure 7:**
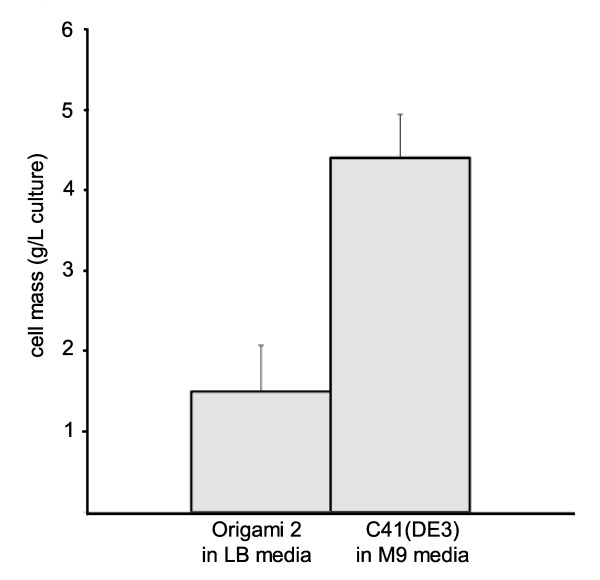
**Mass of isolated cells expressing MBP-6 × His-U24**. Cell masses which correspond to highest yields of protein are notably different between the cell types; although grown in rich LB media, Origami 2 cells (pMAL-c2x-U24) are consistently ~3 × lower in mass than C41(DE3) (pMAL-p2x-U24), which is grown in M9 minimal media. Data is based on mean ± s.d. for n = 4-5 batches of cells isolated from 4 × 1L of culture, expressed at 18°C overnight.

We observed a trend in the amount of protein-expressing cells from large scale preparations of U24, where pMAL-p2x-U24 is expressed in C41(DE3) and pMAL-c2x-U24 in Origami 2 (Figure 7). Despite C41(DE3) being grown in nutrient-limiting M9 media, these cells regularly accumulated to a much higher final mass than Origami 2, which were grown in nutrient-rich LB media. In our hands, Origami 2 cells expressing MBP-6 × His-U24 grew so slowly in M9 media that further attempts with this combination were not attempted (data not shown). It is clear that Origami 2 cells experience extreme metabolic stress compared to other strains like C41(DE3). However, with high yields of U24 protein isolated Origami 2 (Table 1) with low background of other endogenous *E.coli *proteins (Figure 2A&Figure 2B), we hypothesize that the unique metabolism of oxidizing strains like Origami 2 may favor production of recombinant protein while minimizing expression of proteases and other factors which lower the expression of full-length protein.

## Discussion

In the course of expressing and isolating U24, we have shown that membrane protein expression levels can increase when the *E.coli *periplasm or an oxidizing cytoplasm is used in conjunction with the MBP-6 × His dual tag, and that highly pure, soluble membrane protein can be readily obtained in good yields (2-3 mg U24 protein per L of culture, see Table 1). Using this as a model system, we conclude that use of the MBP-6 × His tag, together with low temperature, and either the periplasm of C41(DE3) or oxidizing cytoplasm of strains (Origami 2, SHuffle, etc.) is a worthwhile approach for obtaining other difficult-to-express membrane proteins in *E. coli*. Based on these results, one might expect a several-fold expression level increase over the commonly-used BL21(DE3) cell strain when employing these conditions to certain other membrane protein systems. In the current work, we have also determined that disulfide bonding of the target protein need not be a prerequisite for full-length expression of U24. Ultimately, we utilize the C41(DE3) in M9 media regime to produce isotopically labeled U24 protein for NMR experiments, while reserving the Origami 2 in LB approach to routinely produce unlabeled U24 for all other biophysical studies.

While we are capable of forming (and reducing) a disulfide in our recombinant version of U24 from HHV-6A we must consider the fact that the cysteines are not conserved across the U24 proteins from other viruses, HHV-6B and HHV-7. All three versions of U24 have the ability to sequester cellular receptors in early and intermediate endosomes of human cells [3], yet while U24_HHV-6A _has two cytoplasmic cysteines, U24_HHV-6B _has only one and U24_HHV-7 _has two which are exclusively located in the transmembrane region (and thus are not likely to form disulfide bonds). We cannot rule out whether these differences can affect the activity of U24 on a more precise level, or somehow are involved in regulatory mechanisms that have yet to be discovered.

Using the MBP-6 × His tagging system for a membrane protein of interest with a generic expression strain (i.e.: C41(DE3)), one should quickly be able to test both cytoplasmic and periplasmic expression strategies at high and low temperature. If there is the potential to form a disulfide bond in the membrane protein of interest, yet the oxidation state of the cysteines are unknown, one may benefit from using the reductase deficient SHuffle strain. Should the protein not have any disulfides, DsbC in SHuffle may still act as a chaperone to induce proper folding and avoid unwanted disulfide bonds. If co-overexpression of DsbC should fail to induce native disulfide bonding patterns of an MBP-fusion protein, it may be possible to correct disulfide mispairings by an *in vitro *incubation of MBP-fusions with DsbC [28], or by co-expression with the recently characterized sulfhydryl-oxidase, Erv1p [23].

Impaired disulfide bonding may be but one of several key obstacles in obtaining stable full-length membrane proteins in *E. coli*, especially those from mammalian sources. Polypeptide synthesis is 4-10 times faster in prokaryotes than eukaryotes, and the apparatus by which membrane proteins are inserted into the membrane is different [29]. One approach may be to reduce the rate of synthesis by lowering expression temperature, or to use a weaker promoter. Although MBP appears superior to most other fusion tags in the literature, improvements in yield can result from using other unique tags [30]. Autoexpression media [17] or novel enzyme-based-substrate delivery media (EnBase, [31]) also represents an attractive approach for increasing both cell mass and protein yield. Still, other advances involve the reduction in toxicity associated with membrane protein overexpression [11,25]. While these and other trial-and-error optimization techniques may give a modest improvement in membrane protein yield, increasing our mechanistic understanding of prokaryotic systems of membrane protein translation and folding is clearly warranted. Directed evolution studies [32] and genetic analysis of upregulation, downregulation and mutations [33] made to house-keeping genes [34] during membrane protein expression will undoubtedly continue to have an impact on yields of functional membrane proteins in *E. coli*.

## Methods

### Gene construction

The gene sequence for U24 from HHV-6A was obtained from NCBI [GenBank: Q69559]. Oligonucleotide fragments that represent the full-length U24 gene and primers were synthesized (Nucleic Acid Protein Service Unit, University of British Columbia), replacing codons which are rare in *E. coli *with ones more frequently used. The codon optimized U24 gene was assembled by overlap PCR for insertion into the pMAL-c2x and pMAL-p2x vectors (New England Biolabs). For use with the pMAL-c2x and pMAL-p2x vectors, the amplified gene segment included a *BamHI *site at the 5' end, followed by a sequence coding a hexahistidine tag followed by a thrombin cleavage site (LVPRGS), then the U24 sequence, followed by an amber stop codon then a *HindIII *site at the 3' end. DNA fragments were purified by agarose gel electrophoresis and subsequently extracted using a Qiaquick Gel Extraction Kit (Qiagen).

### Cloning

The isolated gene fragments described above were incubated with Taq polymerase, pCR^®^2.1-TOPO^® ^vector and dATP (all from Invitrogen) and the reaction mixtures were used to transform competent SURE^® ^*E. coli *(Stratagene). The *E. coli *were then plated on Luria Bertani (LB) agar plates containing 50 μg/ml carbenecillin to grow overnight at 37°C. Colonies were selected and subjected to PCR using M13 forward and reverse primers to identify which colonies contained the vector with the correct inserted gene. PCR reactions for colonies which did harbor the gene insert yielded a single band between 500-600 bp when run by agarose gel electrophoresis. These colonies were then grown in 5 ml of LB media with 50 μg/ml carbenecillin overnight and their plasmids were harvested using a QIAprep Spin Miniprep Kit (Qiagen). Isolated plasmid DNA was sent for sequencing (Nucleic Acid Protein Service Unit, University of British Columbia) to confirm that the correct gene sequences were obtained.

The pCR^®^2.1-TOPO^® ^vectors containing the expected U24 gene inserts were digested by the appropriate endonucleases, *HindIII*/*BamHI*. The digested fragments were isolated as previously described, then ligated into the corresponding pre-digested gel-purified pMAL vectors using T4 DNA ligase (Invitrogen). SURE^® ^*E. coli *were transformed with the ligation products, the plasmids were isolated and sequenced as before, and designated pMAL-c2x-U24 and pMAL-p2x-U24.

### Site-directed Mutagenesis

Synthetic primers were purchased (Integrated DNA Technologies) and used according to Quikchange Site Directed Mutagenesis Kit instructions (Stratagene). Vectors pMAL-c2x-U24 and pMAL-p2x-U24 were used as the starting template. To construct the C21S mutant, the primers were 5'-CTGATGATGGACGTTATGTCCGGTCAGGTTTCC-3' and 5'-GGAAACCTGACCGGACATAACGTCCATCATCAG-3', which introduces an Eam1105I-sensitive restriction site. For the C37S mutant, the primers used were 5'-CCTTCGTTGAATCTATTCCGCCACCG-3'and 5'-GGACTGCGGTGGCGGAATAGATTCA-3' and they introduced a *XmnI*-sensitive restriction site. The C21S mutant plasmid was the starting template for constructing the C21SC37S double mutant. All plasmids were isolated and sequenced as described above for wild-type plasmids.

### Small Scale Protein Expression

The expression vector constructs pMAL-c2x-U24 and pMAL-p2x-U24 were used to transform XL1-Blue (Stratagene), BL21(DE3) (Stratagene), C41(DE3) (Lugicen), Origami 2 (EMD Biosciences) and SHuffle (New England Biolabs) *E. coli *strains. Transformed *E. coli *were then plated on LB agar plates containing 50 μg/ml carbenecillin to grow overnight at 37°C (30°C in the case of SHuffle, as per manufacturer's instructions). Single colonies were selected and grown at these temperatures overnight with shaking (225 r.p.m.) in 5 ml of LB, containing 50 μg/ml carbenecillin. Cells were pelleted by centrifugation, then resuspended in 100 ml of fresh medium with antibiotics and again grown for several hours until reaching an OD_600 _= 0.5-1.0. Isopropyl β-D-1-thiogalactopyranoside (IPTG) was added at a final concentration of 0.3 mM and the cells continued to grow for 3 hours at the same temperature (30 or 37°C). For growths carried out at lower temperature, the cultures were cooled in a cold-water bath for 30 minutes before addition of the IPTG, and these cultures were then grown at 18°C for 16-20 hours. All cultures were harvested by centrifugation at 4°C and the cells were stored at -80°C until further use.

### Large Scale Protein Expression

Small starter cultures (5 ml) were set up as described for the small scale expression. In this case, the cultures were grown for only three to five hours before one ml of culture was used to inoculate 100 ml of fresh LB or M9 minimal media containing 100 μg/ml ampicillin. M9 minimal media was supplemented with 50 μg/ml thiamine. These cultures were grown overnight under the same conditions as the 5 ml cultures described in the section above, and then harvested by centrifugation. Cell pellets were resuspended in 4 × 1 L of fresh medium with antibiotics and grown to an OD_600 _= 0.5-1.0. Cultures were cooled in a cold-water bath for 30 minutes before addition of IPTG at a final concentration of 0.3 mM, and they then continued to grow at 18°C for another 16-20 hours. Cultures were harvested by centrifugation at 4°C and the cells were stored at -80°C until further use.

### Protein Extraction and Purification

C41(DE3) cell pellets from 4 L of culture, harboring expressed MBP-6 × His-U24, were thawed on ice and resuspended in lysis buffer (20 mM Na^+^/K^+ ^phosphate, 0.5 M NaCl, pH 7.4), adding DNase I (Roche) and EDTA-free protease inhibitor cocktail (Sigma). Cells were lysed by three passes through a French press, and the lysate was centrifuged for 90 minutes at 25,000 × *g*, 4°C. The pellet was resuspended in solubilization buffer (20 mM Na^+^/K^+ ^phosphate, 0.5 M NaCl, 1% Triton X-100, 10 mM imidazole, pH 7.4), gently stirred for three hours at 4°C, and again centrifuged for 90 minutes at 25,000 × *g*, 4°C. Since substantial amount of target protein was found in both the supernatant and pellet of Origami 2 and SHuffle cells treated with lysis buffer, the lysis buffer step was omitted and these cells were resuspended directly into solubilization buffer with protease inhibitor cocktail prior to being lysed by French press. Supernatants were filtered through a 45 μm filter and loaded on a 5 ml Histrap HP column (GE Biosciences) equilibrated with wash buffer (20 mM Na^+^/K^+ ^phosphate, 0.5 M NaCl, 0.5% Triton X-100, 10 mM imidazole, pH 7.4). The column was washed with 30 column volumes of wash buffer. The imidazole concentration of the wash buffer was raised to 45 mM, and the column was further washed with another 10 column volumes. The MBP-6 × His-U24 protein was eluted from the column in 25 ml of elution buffer (20 mM Na^+^/K^+ ^phosphate, 0.5 M NaCl, 0.5% Triton X-100, 500 mM imidazole, pH 7.4) and dialyzed overnight at 4°C against 1 L of dialysis buffer (20 mM Na^+^/K^+ ^phosphate, 62.5 mM NaCl, 0.5% Triton X-100) using a dialysis membrane with MWCO of 1000 (Spectrum Laboratories).

Dialyzed solution containing the MBP-6 × His-U24 was transferred to a 50 ml polypropylene tube, and 50 units of bovine thrombin (GE Healthcare) were added. The digest was carried out for 48 hours at ambient temperature.

The digest solution was filtered through a 45 μm filter and re-loaded on a 5 ml Histrap HP column equilibrated with dialysis buffer. U24 was collected in the flowthrough, while MBP-6 × His and any undigested MBP-6 × His-U24 were eluted with elution buffer. To remove the thrombin and any trace contaminants that remained, U24 solution was loaded on a 5 ml Q-Sepharose column (GE Biosciences) equilibrated with dialysis buffer and pure U24 was collected in the flowthrough. Purity was assessed by MALDI-TOF mass spectrometry and SDS-PAGE, and protein concentration was measured with the bicinchoninic acid (BCA) assay (Pierce).

### GluC Digests

U24 protein isolated from SHuffle *E.coli *was dialyzed overnight at 4°C against 50 mM Tris·HCl, 100 mM NaCl, pH 7.5 with a 1000 MWCO membrane. *Staphylococcus aureus *Protease V8 (GluC, from New England Biolabs) was dissolved in deionized water to a concentration of 0.1 μg/ml then mixed with U24 (1:17 w/w), ± 20 mM DTT, and incubated for two hours at 25°C. Reactions were quenched by addition of trichloroacetic acid (TCA) at a final concentration of 20% (w/v). Pellets were collected by centrifugation and washed twice with ice-cold acetone then air-dried.

### Far-UV Circular Dichroism

Protein stock solutions of U24 were precipitated by addition of TCA, 10% (w/v), and collected by centrifugation. The protein pellets were then washed with two additions of ice-cold acetone, vortexing and centrifugation. After acetone was removed and the sample air-dried, the protein was reconstituted in 10 mM Tris·HCl, 10 mM NaCl, 10 mM SDS, pH 7.5, +/- 0.5 mM tris(2-carboxyethyl)phosphine (TCEP) with an estimated final protein concentration of 0.11 mg/ml. Protein solutions were sonicated in a water bath until the protein was completely dissolved, then centrifuged briefly to ensure the removal of any particulate matter. Final protein concentrations of the samples were determined by BCA assay.

Spectra were recorded with a J-810 spectropolarimeter flushed with nitrogen gas. Using a cell with a path length of 0.2 cm and a sample compartment that was thermostated to 20°C, samples were scanned at a rate of 50 nm/min with a step size of 1 nm. Spectra were averaged over three scans and corrected for background by subtracting the scans of buffer without protein.

### SDS-PAGE Analysis of Protein Expression

Cell pellets from 0.5 ml of culture were mixed with 50 μl 2 × Novex dye (Invitrogen) supplemented with 5% β-mercaptoethanol and 50 μl of 50% glycerol, vortexed and heated at 95°C for 5 minutes. BenchMark His-tagged molecular weight markers (Invitrogen) and a 5 μl volume of each prepared sample were loaded on a 13% acrylamide gel and run by Tris-Glycine SDS-PAGE. The initial voltage was set to 50 V for one hour, and 100 V for the remainder of the run until the dye front reached the bottom of the gel. Gels were stained with a Coomassie Blue-G250 solution for 1 hour and destained with 50% methanol/10% acetic acid or deionized water alone. For Invision staining, the gels were treated according to manufacturer's instructions (Invitrogen). SDS-PAGE image data was captured using a GelDoc XR imager (Biorad) and densitometric analysis of protein expression was carried out using Quantity One software, V4.6.7 (Biorad). The contour tool and global background subtraction methods were used to select bands and calculate their relative intensities.

Purified U24 samples were mixed with equal volumes of 2 × Novex dye that contained 5% β-mercaptoethanol and then were heated to 95°C for 5 minutes. Samples used in the GluC digest experiment were not heated prior to loading on the gel, and only those that were previously exposed to DTT contained 2.5% β-mercaptoethanol. Gels were run, stained and destained as before, with the exception that a Tris-Tricine gel buffer was used in order to resolve the lower molecular weight proteins and peptides. In these gels, Mark12 molecular weight markers (Invitrogen) were used to estimate protein masses.

### NEM modification

U24 protein isolated from C41(DE3), Origami 2 and SHuffle *E. coli *strains were dialyzed overnight at 4°C against 50 mM Tris·HCl, 100 mM NaCl, pH 7.5 with a 1000 MWCO membrane. N-ethylmaleimide (NEM) was dissolved in the same buffer and diluted to 4 mM. TCEP was dissolved separately in this buffer to a concentration of 20 mM and the pH was made slightly basic by addition of NaOH before diluting further to a TCEP concentration of 2 mM. Samples of U24 were mixed 1:1 (vol:vol) with TCEP solution (+ TCEP samples) or with buffer (- TCEP samples) and incubated for 10 minutes at 37°C. These samples were then mixed 1:1 (vol:vol) with NEM solution (+ NEM samples) or buffer (- NEM samples) and incubated in the dark for 30 minutes at room temperature. Reactions were quenched by addition of trichloroacetic acid (TCA) to a final concentration of 20% (w/v). Pellets were collected by centrifugation and washed twice with ice-cold acetone, and then air-dried.

### MALDI-TOF Mass Spectrometry

Protein samples were dissolved in 50% acetonitrile with 0.1% trifluoroacetic acid. The matrix, sinapinic acid, was dissolved in the same solvent to a concentration of 10 mg/ml. The matrix was spotted on the MALDI target plate followed by the protein solution and another layer of matrix. Volumes added were 1 μL, and the spot was air-dried between each addition. Experiments were performed on Bruker Biflex IV (Bruker Daltonics) MALDI-TOF mass spectrometer, operating in linear ion mode and externally calibrated with horse heart cytochrome C and bovine ubiquitin (Sigma), giving a mass accuracy of ± 0.2%.

## Competing interests

The authors declare that they have no competing interests.

## Authors' contributions

ART performed all experiments reported herein, as well as the data analysis. He wrote the first draft of the manuscript. SKS edited the manuscript. Both authors read and approved the final manuscript.
